# No Negative Priming Effect in the Manual Stroop Task

**DOI:** 10.3389/fpsyg.2019.01764

**Published:** 2019-08-02

**Authors:** Luke Mills, Sachiko Kinoshita, Dennis Norris

**Affiliations:** ^1^Department of Cognitive Science, Macquarie University, Sydney, NSW, Australia; ^2^Department of Psychology, Macquarie University, Sydney, NSW, Australia; ^3^MRC Cognition and Brain Sciences Unit, University of Cambridge, Cambridge, United Kingdom

**Keywords:** negative priming effect, Stroop task, response mode, RT distribution analysis, conflict control

## Abstract

The negative priming effect is an increase in interference when the response to the target on the current trial corresponds to the response to the distractor word on a preceding trial. Contrary to the commonly held belief that the negative priming effect is ubiquitous in the Stroop task, in the original study by Neill (1977), negative priming was found only in the oral, and not the manual Stroop task. The present paper makes three empirical observations. First, we replicate the discrepancy in the finding of the negative priming effect in the oral versus manual Stroop tasks tested under identical conditions, where response mode could be the only the causal factor. Second, we point out that previous manual Stroop experiments reporting the negative priming effect confounded the effect of response repetition. Third, we report the analysis of the negative priming effect at the level of whole RT distribution, which revealed that the effect was absent throughout the RT distribution in the manual task, and it was of constant size across the RT distribution in the oral task. Implications of the results for conflict control in the Stroop task is discussed.

## Introduction

To stay on task while ignoring prepotent conflicting distractors is important in everyday life. A major research tool used to investigate this conflict control process is the Stroop task (Stroop, 1935), in which the participant is presented with a word in color and instructed to name the color, ignoring the word. The finding of an interference effect when the word is incongruent with the response color (e.g., the word GREEN presented in red) relative to a neutral non-readable stimulus (e.g., a row of #s) is highly robust, and is taken as evidence that the word was read, despite the instruction to ignore the word. As noted by Besner (2001), the Stroop interference effect is therefore widely regarded as demonstrating the *automaticity* of word reading; at the same time, however, the size of Stroop interference effect can be modulated, which is taken to indicate attentional *control*.

In a recent review making a case for the *automaticity* of reading in the Stroop task, Augustinova and Ferrand (2014) wrote that “if any intervention is found to indisputably prevent or control word reading, then this finding should be mirrored in complementary analyses, such as those involving negative priming (i.e., an additional indicator of the fact that the word dimension of a Stroop word has been read)” (p. 347). The negative priming effect is the slowdown in response to a stimulus that had to be ignored previously. The effect is well-established in a picture naming paradigm involving two overlapping line drawings presented in different colors (e.g., picture of a sparrow in green superimposed on a picture of a rabbit in red) one of which (e.g., red) designates the to-be-named item (“rabbit” in the present example) (Tipper, 1985). Compared to a control condition in which the preceding trial contains two items that are unrelated to the two pictures in the current trial (e.g., the preceding trial contains a picture of a car in green superimposed on a picture of a tree in red), naming is slowed down when the to-be-named picture in the current trial was the to-be-ignored picture in the preceding trial (e.g., the preceding trial contained a picture of rabbit in green). This effect was originally explained in terms of distractor inhibition in the service of conflict control – “one means by which a response can be directed toward a target stimulus in the presence of a distractor that competes for the control of action, is for inhibition mechanisms to suppress the activation levels of the distractor’s internal representations” (Tipper, 2001, p. 322). While other accounts that do not assume inhibition of the distractor representation have been proposed (see reviews by e.g., Tipper, 2001; Mayr and Buchner, 2007; and also Tipper and Cranston, 1985), in the present context, what is relevant is that the negative priming effect is assumed to be an index of a mechanism of conflict control.

It is widely believed that the negative priming effect is present in the Stroop task. In MacLeod’s (1991) comprehensive review of the Stroop literature, the negative priming effect is listed as one of the “Eighteen Major Empirical Results That Must be Explained by Any Successful Account of the Stroop Effect.” In a more recent review of the Stroop phenomena extending the reach to brain imaging data (MacLeod and MacDonald, 2000) negative priming is described as a well-established phenomenon in the Stroop task. It was a surprise to us, therefore, to read in the classic paper that established the negative priming effect in the Stroop task that the finding was limited to certain task conditions, and it is instructive to describe this study in detail.

Neill (1977) was the first to report finding a negative priming effect in the Stroop task, using the now standard, discrete trial version of the task. Although Dalrymple-Alford and Budayr (1966) have reported the effect earlier using a list version of the (oral) Stroop task, Neill (1977) noted that this finding may have been due to “a tendency to look ahead to the *subsequent* item while trying to respond to the current one” (p. 445). In Experiment 1, Neill used an oral (color naming) Stroop task, with all trials being incongruent^1^ (with four response colors – red, green, blue, and yellow – thus comprising twelve color-word combinations). Eight participants were presented with 1,000 trials (20 blocks of 50 trials), in one 1-hour session.

Neill (1977) classified the trials into *seven* categories on the basis of whether the current target color or distractor matched the distractor or color on the preceding trial (see Table 1 – although Neill (1977) did not use these labels). There were two critical categories: (1) the NONE condition, where there is no overlap between the distractor or target color on the current trial and the preceding trial, e.g., the word YELLOW presented in blue followed by RED presented in green; (2) the WORD-COLOR condition where the distractor word on the preceding trial is the target color on the current trial e.g., the word YELLOW in blue followed by RED in yellow. Neill (1977) defined the negative priming effect as the difference between NONE, which he referred to as the “unrelated” condition, and WORD-COLOR, which he referred to as the “related” condition. (This definition is also standard in the negative priming experiments.) In his Experiment 1, these two conditions yielded mean RTs of 823 ms and 855 ms, respectively (i.e., a 22 ms negative priming effect), a significant difference, with all eight participants showing the effect in the same direction.

**TABLE 1:** Seven categories of trial type with examples based on the relationship between the distractor and response color in the preceding trials and the current trial.

**Trial type**	**Example**
**(Previous trial = YELLOWblue)**	
NONE (“unrelated”)	REDgreen, GREENred
COLOR-COLOR	REDblue, GREENblue
COLOR-WORD	BLUEgreen, BLUEred
WORD-WORD	YELLOWred, YELLOWgreen
WORD-COLOR (“related” or NP)	REDyellow, GREENyellow
COLOR-COLOR-WORD-WORD	YELLOWblue
COLOR-WORD-WORD-COLOR	BLUEyellow

*The color name in CAPS denote the distractor and the color name in lowercase denote the response color (e.g., YELLOWblue denotes the word YELLOW presented in blue). The two last categories are ambiguous: COLOR-COLOR-WORD-WORD is a complete repetition and may be considered to be an instance of COLOR-COLOR or WORD-WORD; COLOR-WORD-WORD-COLOR is a complete reversal and may be considered to be an instance of WORD-COLOR or COLOR-WORD. Each of the five categories except the last two ambiguous categories are expected to occur approximately on one sixth of the trials by chance.*

In Neill’s (1977) Experiment 2, participants responded manually. Six participants were tested over 6 days, with each day containing 20 blocks of 100 trials. On Days 1 and 6, congruent (e.g., the word RED presented in red) and neutral (four 0s presented in color) conditions were included to test if the standard Stroop congruence effects are found (they were: On Day 1, the incongruent, congruent, and neutral conditions yielded mean RT of 727, 665, and 670 ms, respectively; on Day 6, 572, 552, and 557 ms, respectively). In addition, on Days 2–5, the critical 10 color-response blocks alternated with 10 blocks in which participants were instructed to respond to the word. Unlike Experiment 1, this manual Stroop experiment (based on the data from the critical color-response blocks) did not show a negative priming effect: The related (WORD-COLOR) condition was in fact *faster* than the “unrelated” (NONE) condition, 706 ms and 715 ms, respectively.

In a later study, Neill and Westberry (1987) investigated the reason(s) for the discrepancy between the two experiments. They proposed that a likely explanation for the contradictory results of Neill (1977) lies not in the response mode (oral vs. manual), but in the different demands for speed versus accuracy in the two experiments. To test the latter, Neill and Westberry, (2001, Experiment 1) manipulated the instructional emphasis on speed vs. accuracy in a manual Stroop task. The negative priming effect was found when accuracy was emphasized but not when speed was emphasized, which led the authors to conclude that the emphasis on accuracy may have encouraged the use of inhibitory processes. Two points may be noted about this experiment, however. One is that in the “accuracy emphasis” condition under which negative priming effect was found, the RTs were unusually slow (well over 800 ms). The fact that the negative priming effect was absent under the “speed emphasis” condition where the RTs were more representative of manual Stroop experiments could mean that negative priming effect is generally absent in the manual Stroop task conducted under typical experimental conditions. A second point is that this experiment did not compare the oral and manual Stroop tasks and hence the possibility that response mode is a factor responsible for the discrepancy has not been ruled out.

In summary, in the classic study oft-cited as the first report of the negative priming effect, contrary to the popular belief that the negative priming effect is ubiquitous, the original Neill (1977) study did not find an inhibitory negative priming effect in the manual Stroop task. In a later study employing the manual Stroop task, Neill and Westberry (1987) found that negative priming was only present in the manual task when accuracy was emphasized. A recent study using the manual Stroop task (Hazeltine and Mordkoff, 2014) also found no negative priming effect, finding little difference between the NONE condition (684 ms) and the WORD-COLOR condition (690 ms).

In contrast to these null findings, other studies used the manual Stroop task and reported finding a sizable negative priming effect: Besner (2001) reported a 52 ms negative priming effect; Raz and Campbell (2011) reported finding a 20 ms effect (see also Juvina and Taatgen, 2009).^2^ However, negative priming was calculated differently in these studies than in the Neill and Westberry (1987) study. In particular, negative priming was not measured in terms of the difference between the WORD-COLOR condition and the NONE condition, but instead was referenced against a wider range of conditions. We will return to these studies in the Discussion. In the present study, our aim was 2-fold. The first was to see if Neill’s (1977) original findings of inhibitory negative priming effect in the oral Stroop task, but not the manual Stroop task, can be replicated, and the second was to analyze the negative priming effect at the level of whole RT distributions. There were two reasons for conducting this replication study. First, Neill (1977) tested a small number of highly trained participants (8 participants over 1,000 trials in the oral Stroop task and 6 participants in 2,000 trials × 6 sessions = 12,000 trials in the manual Stroop task). It is not known whether the findings can be replicated under more typical experimental conditions. Second, in addition to response mode, Neill’s (1977) two experiments differed in other important ways: The manual experiment was conducted over 6 days, bookended by blocks containing congruent and neutral trials, and further, the color-response blocks alternated with word-reading blocks. Here, the oral and manual Stroop tasks were tested under identical conditions containing the incongruent trials only, hence were the patterns of negative priming effects to differ between experiments, response mode would have to be the only causal factor. Such a result would have important implications for interpreting the data obtained with the manual Stroop task: If the negative priming effect is absent where it is expected, the effect cannot serve as an index of a mechanism of conflict control.

### RT Distribution Analysis

The second aim of our study was to analyze the negative priming effect at the level of whole RT distribution. Previous studies examining the negative priming effect in the Stroop task have analyzed only the mean RT. As pointed out by Balota and Yap (2011), an analysis of RT distributions can provide richer information than the analysis of mean RT, because the distribution of RTs in speeded response tasks is almost always positively skewed, and hence the effect of manipulation may not be captured accurately by the mean. RT distribution analysis could also provide insights into the cognitive mechanism underlying the effect.

The method of RT distribution analysis used in the present study is quantile analysis. Quantile analysis is a non-parametric method of RT distribution analysis that involves rank ordering the RTs for each participant in each condition from fastest to slowest and then dividing them into equal size bins (e.g., the first bin contains the fastest 25% of RTs, the second bin contains the next faster 25% of RTs and so on). The quantiles for each subject in each condition are estimated by taking the mean of the fastest trial of the slower bin and the slowest trial of the faster bin. The quantile estimates are then averaged across each subject in each condition to form the quantile estimates for each condition. The quantile estimates for each condition can then be depicted graphically using a quantile plot and the size of the experimental effect as a function of quantiles can be depicted using a delta plot.

Conflict tasks, such as the Stroop task, have been found to produce three general delta plot patterns (Pratte et al., 2010): In one pattern, the delta slope shows a positive increase across the quantiles, indicating that the size of the effect increases as responses slow. In another pattern, the delta slope is flat, indicating that the size of the effect remains constant across the quantiles. In the third pattern, the delta slope is negative, indicating the size of the effect decreases as responses slow.^3^

Pratte et al. (2010) proposed that the positive and flat delta slope patterns are concordant with evidence accumulation models, such as the diffusion model (Ratcliff, 1978), which view decision making in speeded tasks as a process of accumulating evidence from the stimulus until enough evidence has been accumulated for response selection. In this framework, a positive delta slope is concordant with the manipulation affecting the rate of evidence accumulation (“drift rate” in the diffusion model), while a flat slope is concordant is with a change in decision threshold or “non-decision time” (which subsumes the encoding of stimulus before the evidence accumulation process begins, and the preparation of motor response). It is well-established that both the classic Stroop interference effect as indexed by the difference between the incongruent condition (e.g., GREEN presented in red) and the neutral condition (e.g., a row of #s presented in red) and the Stroop congruence effect as indexed by the difference between the incongruent condition and the congruent condition (e.g., GREEN presented in green) increase as responses slow, i.e., they show a positive delta slope (e.g., Steinhauser and Hübner, 2009; Pratte et al., 2010), and this is the case for both the oral and manual Stroop task (e.g., Kinoshita et al., 2017). This positive delta slope may be interpreted as reflecting that the evidence needed for response selection is accumulated from the word distractor at the same time as the color target, and the two are integrated during the evidence accumulation process. On the assumption that the classic Stroop interference effect (and the Stroop congruence effect) and the negative priming effect have the same origin, in inhibitory control, it would be expected that the negative priming effect also shows a positive delta slope (i.e., an increase in the effect as responses slow).

## Experiment 1 (Oral)

### METHOD

#### Participants

Twenty students from Macquarie University participated in the experiment in return for course credit. Both experiments reported here were approved by the Macquarie University Human Research Ethics Committee.

#### Design

Experiment 1 used an oral Stroop color naming task. The dependent variables were color response latency and error rate, examined as a function of the five types of relationship between the distractor and response color on the preceding and current trials described in the Introduction (see Table 1).

#### Materials

The stimuli were four color names, RED, YELLOW, GREEN and BLUE presented in one of four colors, red (RGB 255, 000, 000), yellow (RGB 255, 255, 000), green (RGB 000, 128, 000) or blue (RGB 000, 000, 255), against a gray background (RGB 200, 200, 200). Each word was presented only in an incongruent color (e.g., RED was presented in yellow, green and blue, but not in red) thus there were twelve color-word combinations in total.

Each color-word combination was presented 32 times, resulting in 384 trials. The 384 trials were divided into eight sublists of 48 trials with each sublist containing an equal number of the 12 color-word combinations. Different random order of trials was generated for each sublist.

#### Apparatus and Procedure

Participants were tested individually, seated approximately 60 cm in front of a flat screen monitor, upon which stimuli were presented. Each participant completed 384 color identification trials, presented in eight blocks (with each block containing 48 trials) with a self-paced break between the blocks. A practice block of 48 trials containing an equal number of color-word combinations preceded the test blocks.

Participants were instructed at the outset of the experiment that on each trial they would be presented with a word presented in an incongruent color, in one of four colors, red, yellow, green or blue. The participants were instructed to make their responses as quickly as possible, while still maintaining accuracy. Before the experiment, participants were given eight color naming practice trials with five hash signs (#####) presented in each of four response colors.

Stimulus presentation and data collection were achieved using the DMDX display system developed by KI Forster and JC Forster at the University of Arizona (Forster and Forster, 2003). Stimulus display was synchronized to the screen refresh rate (10.01 ms).

Each trial started with the presentation of a fixation signal (a plus sign) for 250 ms, in the center of the screen. It was replaced by a blank screen for 50 ms, then by a word presented in one of four colors (red, yellow, green or blue) for a maximum of 2,000 ms, or until the participant made a response. In the oral Stroop task, the participant spoke the color name into the microphone which triggered the voice key. After the participant’s response, the screen went blank for 816 ms after which the next trial started. All stimuli were presented in Lucinda Console 12 point font. Participants were given no feedback during the experiment. The experimenter sat next to the participant and recorded errors during the experiment.

### Results

Two sets of analyses are reported below. The first analysis is of individual trial RTs, using linear mixed effect model (Baayen, 2008). Next, we analyzed for the negative priming effect at the level of the RT distribution using quantile analysis and delta plots.

### Mean RT

In this and subsequent experiment, correct RTs and error rates were analyzed according to the following procedure. In the analysis of RTs, we first examined the shape of the RT distribution for correct trials, and excluded those faster than 250 ms as outliers (most of the fast outliers were voice key trigger errors). In Experiment 1, 282 data points (out of 7501 trials, 3.7%) were identified as outliers.

We analyzed the RT data using linear mixed effects model with Trialtype (NONE, COLOR-COLOR, COLOR-WORD, WORD-WORD, WORD-COLOR) as a fixed factor and subjects and stimuli as crossed random factors (Baayen, 2008). (The additional ambiguous categories were included, but were not considered, in the analysis.) RT was log-transformed to reduce the positive skew as recommended by Baayen (2008). LogRT was analyzed using the *Lme4* package (Version 1.1-5 Bates et al., 2014), implemented in R Version 3.4.3 (R Core Team, 2017). Degrees of freedom (Satterthwaite’s approximation) and *p*-values were estimated using the *lmerTest* package (Version 2.0-33 Kuznetsova et al., 2016). The initial model included only the random intercepts on participants and stimuli, and if the model comparison indicated a significantly better fit, the more complex model including random slopes (of the Trialtype factor) was preferred.

Error rates were analyzed with generalized linear mixed effects model with subjects and stimuli as crossed random factors, using the logit function appropriate for categorical variables (Jaeger, 2008). In both experiments, the model tested was: Error rate ∼ Trialtype + (1 | subject) + (1 | stimulus), with the Trialtype factor referenced to the NONE condition.

The mean correct RT and error rates are shown in Table 1.

In Experiment 1, the final model we report is: logrt ∼ Trialtype + (1| stimuli) + (1 + Trialtype| subject), with the Trialtype factor referenced to the NONE condition. The model showed that WORD-COLOR condition was significantly slower than the NONE condition, *B* = 0.057, SE = 0.010, *t* = 5.509, *p* < 0.001, i.e., a negative priming effect (32 ms). The COLOR-COLOR condition was significantly faster than the NONE condition, *B* = −0.097, SE = 0.016, *t* = −5.951, *p* < 0.001, i.e., a response repetition benefit (66 ms). The WORD-WORD condition was marginally slower than the NONE condition (13 ms), *B* = 0.026, SE = 0.012, *t* = 2.133, *p* < 0.05. The COLOR-WORD condition did not differ significantly from the NONE condition (10 ms), *B* = 0.022, SE = 0.012, *t* = 1.803, *p* = 0.085. We also computed Bayes Factors (BF) using the BayesFactor R package (Morey and Rouder, 2015). A BF indexes the relative strength of evidence for one hypothesis over another. The typical value considered to be reliable evidence for a hypothesis is a BF > 3 (Jeffreys, 1961). The BF for the negative priming effect was 38,917,899, indicating exceedingly strong evidence for its presence.

### Error Rate

Error rate was not analyzed as it was too low to warrant an analysis.

### RT Distribution Analysis

**FIGURE 1:**
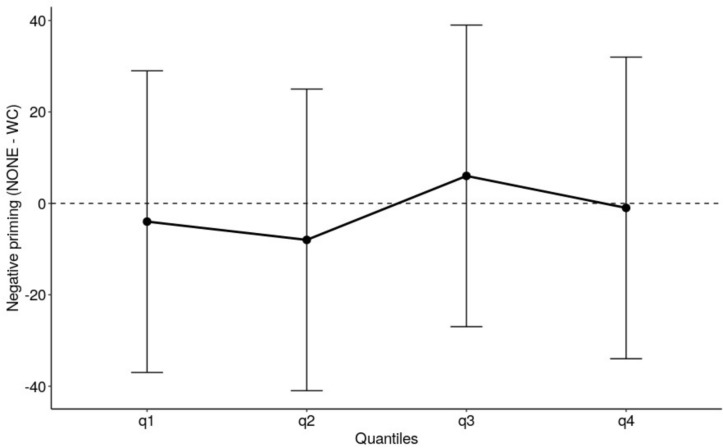
The delta plot depicts the size of the negative priming effect in Experiment 1 oral Stroop task. The error bars are 95% confidence intervals.

The quantiles for the negative priming conditions (NONE vs. WORD-COLOR) were estimated using QMPE version 2.0 (Heathcote et al., 2004). This involved rank ordering the correct RTs for each subject in both the NONE and WORD-COLOR conditions from fastest to slowest and then dividing them into four equal sized bins (i.e., the first bin contains the fastest 25% of RTs, the second bin contains the next fastest 25% of RTs, etc.). The average of the slowest trial of the faster bin and the fastest trial of the slower bin made the 4 quantile estimates. The quantiles were analyzed using a 2 (negative priming: NONE vs. WORD-COLOR) X 4 (quantiles) ANOVA in JASP version 0.9 (JASP Team, 2018). Averaged across quantiles the negative priming effect was significant, *F*(1,19) = 15.6, *p* < 0.001, with RTs for WORD-COLOR trials being slower than for NONE trials. The negative priming effect did not interact with quantiles, *F*(3,57) = 0.5, *p* = 0.693, indicating that the magnitude of the negative priming effect was constant across the quantiles of the RT distribution, resulting in a flat delta slope (see Figure 1).

## Experiment 2 (Manual)

### Participants

Twenty-one students from Macquarie University, additional to those in Experiment 1, participated in the experiment in return for course credit.

### Design and Materials

The design and materials were identical to Experiment 1.

### Apparatus and Procedure

The apparatus and the general procedure were identical to those of Experiment 1, except that the response mode was manual. Participants were instructed that they will be presented with stimuli consisting of color names presented in an incongruent color, and their task was to identify the color of the stimulus, as quickly and accurately as possible, by pressing one of four keys. The participants were instructed to make their responses as quickly as possible, while still maintaining accuracy. They were instructed to press the key Z for red, X for yellow, N for green, and M for blue (the four keys occurred in the bottom row of the QWERTY keyboard), the Z and X keys with their left middle and index fingers, and the N and M keys with their right index and middle fingers. During the practice block a card showing the spatial arrangement of the response keys colored in the corresponding color was displayed to facilitate learning the key assignment; the card was removed at the beginning of the test trials.

Participants were given a feedback following each trial (the word “CORRECT” or “WRONG” presented during the intertrial interval).

### Results

The same procedure for the preliminary treatment of RT data as Experiment 1 was applied to Experiment 2. In this experiment, out of a total of 7446 data points, no data point was identified as an outlier (faster than 250 ms).

#### Mean RT

The final model we report is: Logrt ∼ Trialtype + (1| stimuli) + (1 + Trialtype| subject), with the Trialtype factor referenced to the NONE condition. In this experiment, there was little difference between the WORD-COLOR condition and the NONE condition (−2 ms), *B* = −0.011, SE = 0.015, *t* = −0.731, *p* = 0.47, i.e., no negative priming effect. As in Experiment 1, the COLOR-COLOR condition was significantly faster than the NONE condition, *B* = −0.32, SE = 0.027, *t* = −12.353, *p* < 0.001, i.e., a response repetition benefit (208 ms). The WORD-WORD condition did not differ from the NONE condition, *B* = −0.027, SE = 0.014, *t* = −1.908, *p* = 0.068. The COLOR-WORD condition did not differ from the NONE condition, *B* = −0.022, SE = 0.016, *t* = −1.395, *p* = 0.17. As in Experiment 1, we calculated the BF for the negative priming effect. Here, it was 0.08 for the presence of the effect (i.e., 13 for the null effect) indicating strong evidence for the *absence* of negative priming effect.

#### Error Rate

The only condition to differ from the NONE condition was the COLOR-COLOR condition, *B* = 0.8403, SE = 0.1605, Z = −5.236, *p* < 0.001.

#### RT Distribution Analysis

**FIGURE 2:**
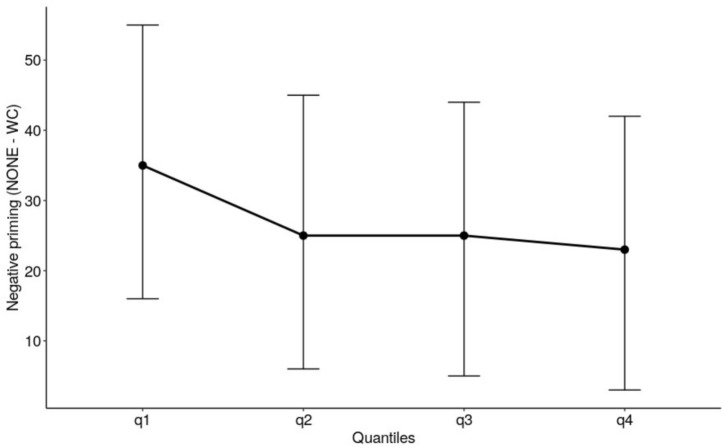
The delta plot depicts the size of the negative priming effect in Experiment 2 manual Stroop task. The error bars are 95% confidence intervals.

The quantile analysis in experiment 2 used the same procedure as in experiment 1. Averaged across quantiles there was no negative priming effect, *F* (1, 20) = 0.020, *p* = 0.888, with there being no significant difference between RTs for WORD-COLOR trials and NONE trials. There was no negative priming effect across the quantiles of the RT distribution, *F*(3,60) = 0.209, *p* = 0.890. The delta slope for the negative priming effect was flat and not significantly different from 0 (see Figure 2).

## General Discussion

The results of Experiments 1 and 2 are straightforward: While an inhibitory negative priming effect (32 ms) was found in the oral Stroop task, there was no hint of the effect in the manual Stroop task. This pattern replicates the original pattern reported by Neill (1977) under a more typical experimental condition. The absence of an (inhibitory) negative priming effect in the manual task is also consistent with the recent result reported by Hazeltine and Mordkoff (2014).

As noted in the Introduction, in contrast to these null results, a couple of studies used the manual Stroop task and reported finding sizeable negative priming effects (Besner, 2001; Raz and Campbell, 2011). Besner (2001, Experiment 3) presented Stroop stimuli in which only a single letter was colored, and in an experiment in which 80% of the trials were incongruent and 20% were congruent, the Stroop congruence effect was minimal (1 ms). Despite the absence of the Stroop congruence effect, Besner reported finding a large (52 ms) negative priming effect. Raz and Campbell (2011) used an equal proportion of congruent, incongruent and neutral trials, and reported that in high-hypnotizable participants a post-hypnotic suggestion for word blindness (that the words would appear “gibberish”) reduced the Stroop congruence effect, but it did not impact on the size of negative priming effect (20 ms with the word blindness suggestion present and absent). Augustinova and Ferrand’s (2014) call to investigate the negative priming effect was made with these studies in the background, with the dissociative effects of single letter coloring manipulation and/or word blindness suggestion on the Stroop congruence effect and the negative priming effect as a theoretical puzzle that needs to be solved.

However, a closer look at these studies suggests that how the negative priming effect was calculated is not the same as Neill (1977). Specifically, Besner (2001) wrote “All incongruent trials in which a stimulus was preceded by an incongruent trial on which the response was correct were classified either as *related*, in which case the irrelevant word on the previous trial was the same as the color on the current trial, or as *unrelated*, in which case the irrelevant word on the previous trial was different from the color on the current trial.” (p. 327). From this description, it appears that while the definition of the “related” condition is the same as that described by Neill (1977) corresponding to the WORD-COLOR condition here, Besner’s “unrelated” condition seems to have included all other conditions (i.e., COLOR-COLOR, WORD-WORD, etc.), not just the NONE condition. This is also the case with Raz and Campbell (2011). They defined as the “NP (negative priming)” trial those in which two consecutive trials were incongruent and the distractor word on the preceding trial matched the target color of the current trial, and as CTRL (control) trials “an incongruent trial pair wherein the word ignored in the first trial was different from the ink color of the immediately following trial” (p. 313). It is apparent from the examples shown in their Figure 1 (p. 314) that the CTRL condition included not only the NONE condition, but also the WORD-WORD condition (and though not shown in the example, their definition could also include the other conditions like COLOR-COLOR, and COLOR-WORD). The fact that Raz and Campbell noted that there were almost three times as many CTRL trials (2691) as the NP trials (1021) suggests that the CTRL trials were not the same as Neill’s “unrelated” (“NONE”) trials, because the frequency of “related” and “unrelated” trials, expected by chance, should be roughly equal.

If it is the case that Besner, 2001 and Raz and Campbell (2011) defined the “unrelated” (or control) condition as all conditions other than the “related” (WORD-COLOR) condition, this would explain why these studies reported finding a “negative priming effect” in a manual Stroop task. In the present experiments, for both oral and manual, the COLOR-COLOR condition was substantially faster than all other conditions. Thus, including all conditions other than the WORD-COLOR (“related”) condition as the comparison condition would result in a large difference (see Table 2, the last row). The “negative priming effect” reported by Besner (2001) and Raz and Campbell (2011) may have reflected this benefit due to repeating the response to the target. This is orthogonal to the target’s relationship to the distractor, and consequently has little to say about the resolution of conflict.

**TABLE 2:** Mean Color Response Latencies (RT, in ms) and Percent Error Rates (%E) in Experiment 1 (Oral) and Experiment 2 (Manual).

		**Response mode**			
		**Oral**		**Manual**	
**Trial type**	**Example**	**RT**	**%E**	**RT**	**%E**
**(Previous trial = YELLOWblue^a^)**					
NONE	REDgreen	637	3.7	763	9.4
COLOR-COLOR	REDblue	571	2.4	555	4.7
COLOR-WORD	BLUEgreen	647	0.9	740	7.8
WORD-WORD	YELLOWred	650	2.1	743	8.8
WORD-COLOR	REDyellow	669	3.0	761	8.9
Negative priming effect (WORD-COLOR – NONE)		32	−0.7	−2	−0.5
Response repetition benefit (NONE – COLOR-COLOR)		66	1.3	208	4.7
WORD-COLOR – all other		168	1.6	61	1.3

*^*a*^The color name in CAPS denote the distractor and the color name in lowercase denote the response color (e.g., YELLOWblue denotes the word YELLOW presented in blue).*

The present study also analyzed the negative priming effect in terms of the whole RT distribution. For the manual Stroop task, this analysis corroborated the analysis of mean RT and showed that the negative priming effect was absent throughout the whole RT distribution. For the oral Stroop task, the negative priming effect showed a flat delta slope i.e., the effect remained constant throughout the RT distribution. It is of interest to note that this pattern is different from the classic Stroop interference effect and the Stroop congruence effect which have consistently been shown to increase as responses slow, i.e., a positively sloped delta plot (e.g., Pratte et al., 2010), which is interpreted in terms of the *rate* of evidence accumulation (“drift rate” in the diffusion model terms). More specifically, the information that determines response selection (what color is it?) is accumulated from the word distractor as well as the color target and integrated during the evidence accumulation process, with the conflicting (incongruent) information reducing the rate of evidence accumulation. The fact that the negative priming effect, in contrast, showed a flat delta slope suggests that unlike the Stroop interference effect or the Stroop congruence effect, the origin of the negative priming effect is not in the evidence accumulation process. It is relevant to note in this regard that Neill and Westberry (1987) reported that (under the accuracy emphasis in the manual Stroop task) the negative priming effect (which they referred to as the “distractor suppression effect”) was found also with neutral trials (consisting of a series of 0s) as well as the incongruent trials. That is, response on the current trial was slowed when it matched the response that would have been required to the distractor in the previous trial, even when the stimulus in the current trial contained no conflicting information. This suggests that the negative priming effect does not reflect a mechanism of control that attempts to reduce informational conflict, consistent with our interpretation that the negative priming effect does not reflect the conflict in the evidence (information) accumulation process. Further, it is contrary to the suggestion by Tipper (2001) cited in the introduction to our paper, that negative priming reflects the inhibition of the distractor’s internal representation. Our view is consistent with Neill and Westberry’s (1987) own interpretation that the negative priming effect does *not* reflect the inhibition of the activated representations themselves, but instead reflects the suppression of “access to overt responses”, which is an idea first proposed by Tipper and Cranston (1985). In other words, it is not the informational conflict from the distractor word that is suppressed, it is the naming response that is suppressed.

An important question is why is negative priming a robust finding in the oral Stroop task but is only found under a narrow range of conditions in the manual Stroop task. A point of difference between the two tasks is that only the oral task requires a speech response. As noted by Roelofs (2003), in alphabetic systems, written words are intrinsically linked with their sounds. In contrast, words are not linked with a specific key on the keyboard. Perhaps the arbitrary nature of the color-key mapping in the manual task reduces the strength of response conflict caused by the distractor, which in turn reduces the need for the response to the distractor to be suppressed. We hope that this paper leads to further investigation of this possibility.

## Conclusion

The present study made three empirical contributions. First, we replicated the absence of the negative priming effect in the manual Stroop task when the effect was found in the oral Stroop task tested under identical conditions. Second, we pointed out that previous manual Stroop experiments reporting the negative priming effect confounded the effect of response repetition. Third, we reported the analysis of the negative priming effect at the level of whole RT distribution, which revealed that the effect was absent throughout the RT distribution in the manual task, and it was of constant size across the RT distribution in the oral task. This pattern contrasts sharply with the pattern of Stroop interference effect and the Stroop congruence effect. We take these findings to argue that the negative priming effect does not serve as an index of control of informational conflict in the Stroop task.

## Data Availability

The datasets generated for this study are available on request to the corresponding author.

## Ethics Statement

The studies involving human participants were reviewed and approved by Faculty of Human Sciences Human Research Ethics Sub-Committee. The patients/participants provided their written informed consent to participate in this study.

## Author Contributions

LM, SK, and DN made substantial contributions to the conception and design of the work, acquisition, analysis, and interpretation of data for the work, drafting and editing of the work, approves of the work being published and agreed to be accountable for all aspects of the work.

## Conflict of Interest Statement

The authors declare that the research was conducted in the absence of any commercial or financial relationships that could be construed as a potential conflict of interest.

## Footnotes

1 It is worth noting that in the picture naming task standardly used to investigate the negative priming effect, the overlapping pictures are always different hence the trials are all incongruent.

2 Juvina and Taatgen (2009) used an unusual response procedure. In their study, there were four response colors, but only two response keys. On each trial the two response options indicating color names (presented in black), one of which corresponding to the correct response color, were shown, one of the left and one on the right on the screen. This response procedure differs substantially from the standard response procedure in the manual Stroop task in which each response color is assigned a key, and the key assignment remains constant throughout the experiment. As pointed out by Juvina and Taatgen themselves, their response procedure places a greater requirement to read the word than the standard procedure, and it is unclear whether the sizable negative priming effect (45 ms) they reported was due to the non-standard response procedure, or the way the negative priming effect was calculated (see section “General Discussion”), and this study will not be discussed further.

3 The negative delta slope pattern is unusual in conflict tasks, and has been found only with some versions of the Simon and Flanker tasks. The readers are referred to De Jong et al. (1994), Pratte et al. (2010) and Burle et al. (2014) for detail.
